# Therapeutic efficacy and safety of botulinum toxin A injection in plantar fasciitis: A systematic review and meta-analysis

**DOI:** 10.1371/journal.pone.0312908

**Published:** 2024-12-17

**Authors:** Qian Li, Jing Zhang, Jie Sun, Chengfei Gao, Jing Zhao

**Affiliations:** 1 Department of Rehabilitation Medicine, Zibo Central Hospital, Zibo, Shandong, China; 2 Department of Rehabilitation Medicine, The Affiliated Hospital of Qingdao University, Qingdao, Shandong, China; Lokman Hekim University, TÜRKIYE

## Abstract

**Objective:**

The purpose of this meta-analysis was to evaluate the therapeutic efficacy and safety of botulinum toxin A (BTA) injections for treatment of plantar fasciitis (PF).

**Methods:**

This review adhered to the PRISMA guidelines, conducting a comprehensive search of the PubMed, Web of Science, EMBASE, and Cochrane Library databases for eligible studies from their inception to December 30 2023. The inclusion criteria were limited to randomized controlled trials (RCTs) comparing BTA injections with control interventions in terms of pain reduction, functional improvement, or the occurrence of adverse events in treating patients with PF were extracted for meta-analysis. Relevant data were extracted using an electronic spreadsheet and analyzed with Stata 16.0 software. The quality of included studies was assessed using the Cochrane Collaboration’s tool.

**Results:**

A total of 655 studies were retrieved and subsequently screened. Seven RCTs, comprising 305 participants, met the eligibility criteria and were included in the meta-analysis. The pooled results indicated that BTA injections led to significant pain reduction only at 1-month posttreatment (SMD = -1.72, 95% CI [-3.10, -0.34], p = 0.01]) and sustained functional improvement over twelve months (SMD = 25.10, 95% CI [9.67, 40.53], p = 0.001) compared to the control group. There was no significant difference in the occurrence of adverse events between the BTA and control interventions (OR = 0.16, 95% CI [-1.00, 1.32], p = 0.79).

**Conclusion:**

This meta-analysis suggested that BTA injection could be an effective and safe therapeutic strategy for plantar fasciitis. However, further larger-scale, rigorously designed RCTs are needed to validate these findings and determine the optimal injection dosage and site for BTA in the treatment of plantar fasciitis.

## Introduction

Plantar fasciitis (PF) is a degenerative condition of the plantar fascia that affects approximately 10% of the general population [1]. It is characterized by heel pain at the calcaneum origin of the plantar fascia as a result of excessive strain and constant microtrauma on the fascia leading to an inflammatory reaction [2, 3]. Hence, therapy for plantar fasciitis focuses on reducing the muscle tightness that initiates the injury or eliminating the inflammation that exacerbates the injury [4].

Generally, a combination of conservative interventions, including nonsteroidal anti-inflammatory medications, stretching exercises, orthotic devices, and extracorporeal shock-wave therapy, is recommended as the first-line treatment for plantar fasciitis [5–8]. However, about 10% of patients experience poor outcomes with conservative treatments. If symptoms persist despite conservative approaches, invasive procedures such as dry needling, miniscalpel-needle therapy, and various local injections may be considered [9].

Corticosteroid infiltration has long been a commonly used treatment for patients with PF. However, this treatment modality is known to provide only short-term pain relief and is often associated with undesirable adverse effects, especially in case of multiple injections [4, 10]. Another injectable option is botulinum toxin type A (BTA). Benefiting from its efficacy in pain control and muscle relaxation, BTA has been utilized in the treatment of various chronic musculoskeletal diseases, particularly in refractory conditions [11, 12]. Recently, numerous clinical studies have examined the effects of BTA injections in the management of PF. Most studies reported positive effects of BTA on pain relief and functional improvement [13–15], while others studies presented conflicting results [16, 17]. Therefore, we systematically searched and analyzed all published randomized controlled trials (RCTs) available to critically assess the efficacy and safety of BTA injection in the management of plantar fasciitis.

## Methods

This systematic review adhered to the Preferred Reporting Items for Systematic Reviews and Meta-Analyses (PRISMA) guidelines [18]. The protocol for this review was registered with the International Prospective Register of Systematic Reviews (PROSPERO) (Registration number: CRD42023467697).

### Eligibility criteria

The inclusion criteria were formulated according to the PICOS principle: 1) Population: adult patients diagnosed with plantar fasciitis; 2) Intervention: any form of intramuscular or subcutaneous injections of BTA; 3) Comparison: patients in the control group receiving placebo or conventional treatments; 4) Outcomes: the change in pain before and after treatment was considered the primary outcome, with secondary outcomes including functional scores and the occurrence of adverse events (AEs). 5) Study designs: randomized controlled trials.

The exclusion criteria were as follows: 1) studies involving patients with a prior treatment history; 2) studies missing pain, functional and AEs data for statistical analysis; 3) animal studies, case reports, reviews, conference papers, and other non-RCTs.

### Search strategy

Electronic databases, including PubMed, Web of Science, EMBASE, and Cochrane Library, were searched for eligible studies from database inception to December 30 2023. The search strategy employed a combination of MeSH terms and keywords related to “plantar fasciitis,” “chronic plantar fasciitis,” “chronic heel pain,” “plantar fasciopathy,” “Botulinum toxin type A,” “BoNT-A,” “BTA,” “randomized controlled trial”, “randomized clinical trial”, and “RCT”. This strategy was adapted for each database, with no language restrictions applied. A manual search was also conducted on the references of all included studies and relevant reviews to identify additional eligible studies.

### Study screening and selection

All search results were imported into EndNote software for data screening and duplicate removal. Two authors (Q.L. and J.S.) independently screened study titles and abstracts based on predefined eligibility criteria. A consensus meeting was held when disagreements arose, and a third reviewer (J.Z.) was consulted if necessary.

### Data extraction and outcome measures

An electronic spreadsheet was used to collect the following data from all included studies: first author, year of publication, study design, number and demographics of participants, details of the interventions, outcomes, and follow-up. The differences in pain and functional assessments as well as the occurrence of adverse events between the interventions were evaluated. Two reviewers (Q.L. and J.S.) independently extracted the data, and any disagreement between them was resolved through consensus with the involvement of a third author (J.Z.).

### Study quality assessment

Two reviewers (Q.L. and J.S.) used “The Cochrane Collaboration’s tool for assessing risk of bias” to appraise the quality of the included studies [19]. The tool evaluated seven dimensions of bias: random sequence generation, allocation concealment, blinding of participants and personnel, blinding of outcome assessment, incomplete outcome data, selective reporting, and other bias. Reviewers’ judgments on each dimension were categorized as “low risk,” “high risk,” or “unclear risk” of bias. A third reviewer (J.Z.) was involved if two reviewers disputed the results.

### Statistical analysis

Statistical analyses were conducted using Stata 16.0 (StataCorp LP, College Station, TX). For continuous outcomes using the same assessment scale, we pooled weighted mean difference (WMD) to report treatment effect; for those with different scales, the standardized mean difference (SMD) was calculated. The I^2^ statistic measured heterogeneity among the studies, with an I^2^ value >50% signaling significant heterogeneity and prompting the use of a random-effects model, while an I^2^ <50% warranted a fixed-effect model [20]. Forest plots visually represented result differences between the BTA and control groups across all included studies. Where possible, a funnel plot was generated to assess potential publication bias among the studies [21]. A p-value <0.05 was deemed statistically significant.

## Results

### Study selection process

**Fig 1:**
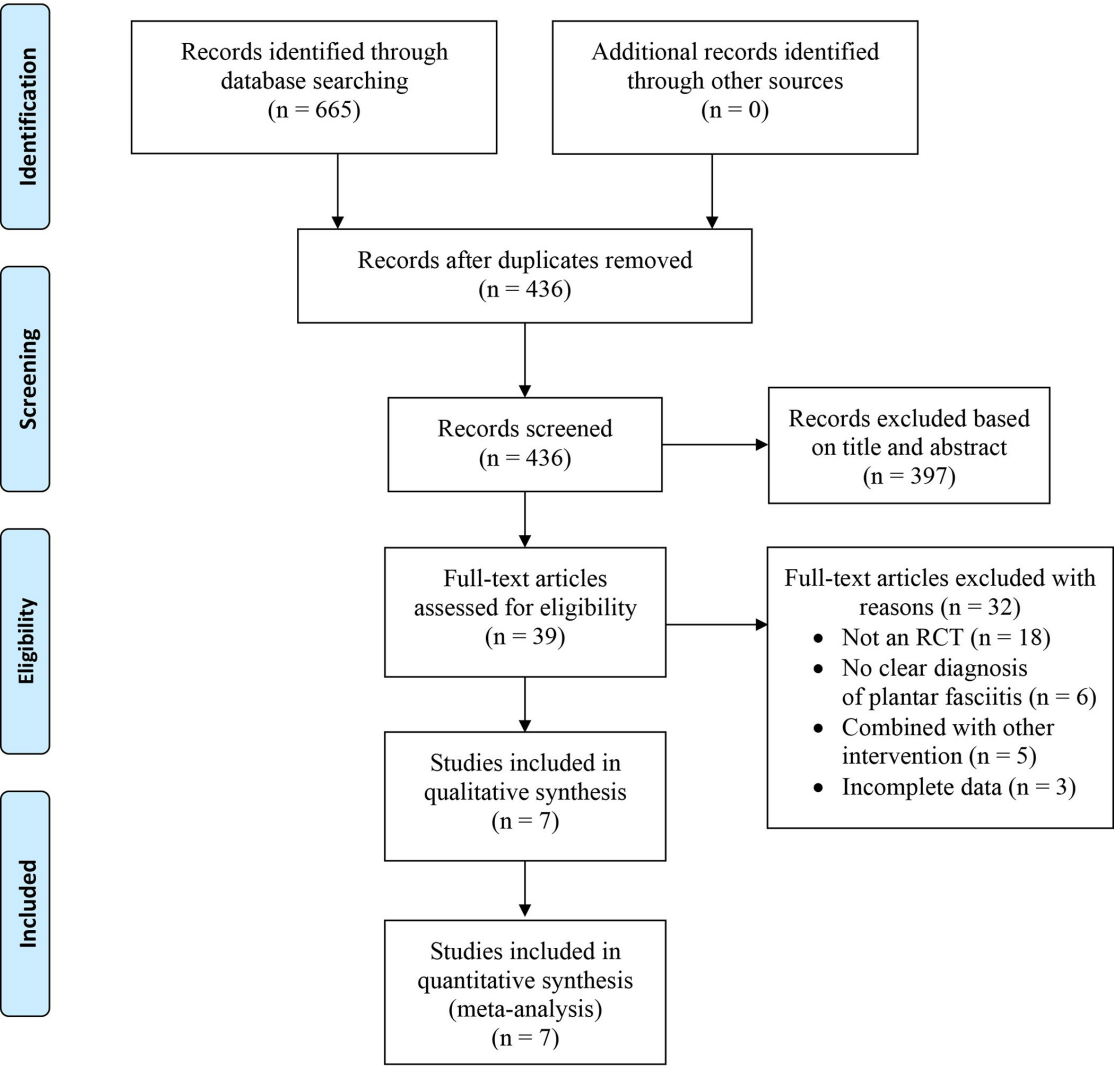
Flow diagram of the study selection process based on PRISMA guideline.

After the literature search, a total of 655 potentially eligible articles were retrieved. Following the removal of duplicate records, 436 studies were screened, with 374 excluded after assessing their titles and abstracts. Subsequently, 39 full-text articles were reviewed for possible eligibility and 32 were excluded for the following reasons: not being RCTs (18), lacking a clear diagnosis of plantar fasciitis (6), being combined with other interventions (5), and having incomplete data (3). See S1 Table for detail. Finally, seven studies [13, 14, 16, 17, 22–24] met all eligibility criteria and were included in this meta-analysis. The PRISMA flowchart was applied to illustrate the step-by-step selection process (**Fig 1**).

### Characteristics of included studies

A total of 305 patients with plantar fasciitis were included in this systematic review and meta-analysis. Patients’ ages ranged from 21 to 77 years, with more females than males (214/91). The dosages of injected BTA among included studies ranged from 50U to 200U, diluted in 0.7–2.5mL of normal saline. BTA were injected into the origin of the plantar fascia in most studies [13, 14, 16, 17, 23], whereas two others selected gastrocnemius as the injection target [22, 24]. All studies performed BTA or placebo injections only once, administered under ultrasound or electromyography guidance. Follow-up periods in the studies varied from 4 weeks to 12 months. The main characteristics of the included studies were summarized in **Table 1**.

**Table 1:** The characteristics of included studies.

Author/Year	Study design	Randomization method	Sample size	M/F	Age (Int vs. Con)	Intervention group (n)	Control group (n)	Guidance of BTA injection	Site of injection	Outcome measurements	Follow-ups
Abbasian et al., 2020	RCT	Random number table	N = 28	(10F/18M)	47.3±6.1 vs. 45.6±9.7	BTA 70U (1.5mL) (n = 15)	Normal saline (1.5mL) (n = 13)	Ultrasound guidance	Medial head of the gastrocnemius	VAS, AOFAS, ROM	Baseline, month 1, 3, 6, 12
Ahmad et al., 2016	RCT	Computer-generated	N = 50	(36F/14M)	48.6 (33–61) vs. 51.3 (31–69)	BTA 100U (1.0mL) (n = 25)	Normal saline (1.0mL) (n = 25)	Electromyographic guidance	Origin of the plantar fascia and tender region	VAS, FAAM	Baseline, month 6, 12
Babcock et al., 2005	RCT	Computer-generated	N = 27	(18F/9M)	44(21–65)	BTA 70U (0.7mL) (n = 22)	Normal saline (0.7mL) (n = 21)	Not reported	Origin of the plantar fascia and tender region	VAS, MFS	Baseline, week 3, 8
Huang et al., 2010	RCT	Not reported	N = 50	(38F/12M)	54.4±9.6 vs. 51.5±5.5	BTA 50U (1.0mL) (n = 25)	Normal saline (1.0mL) (n = 25)	Ultrasound guidance	Origin of the plantar fascia	VAS, Plantar fascia thickness, Fat pad thickness	Baseline, week 3, 12
Peterlein et al., 2012	RCT	Computer-generated	N = 40	(32F/8M)	52.4 vs. 51.8	BTA 200U (2.0mL) (n = 20)	Normal saline (2.0mL) (n = 20)	Not reported	Origin of the plantar fascia	VAS, PPT, ROM, Adverse events	Baseline, week 2,6,10, 14, 18
Roca et al., 2016	RCT	Computer-generated	N = 72	(53F/19M)	54.4±13.3 vs. 50.4±9.5	BTA 100U (1.0mL) (n = 36)	ESWT (n = 36)	Not reported	Origin of the plantar fascia	VAS, plantar fascia thickness	Baseline, month 1
Ruiz-Hernández et al., 2024	RCT	Excel	N = 38	(27F/11M)	51±2vs. 56±2	BTA 200U (2.5mL) (n = 20)	Stretching (n = 16)	Ultrasound-guided	Medial and lateral gastrocnemius, perifascial location	VAS, AOFAS, plantar fascia thickness	Baseline, month 1, 3, 6, 12

RCT: Randomized Controlled Trial; F: Female; M: Male; Int: Intervention Group; Con: Control Group; BTA: Botulinum Toxin A; ESWT: Extracorporeal Shock Wave Therapy; VAS: Visual Analogue Scale; AOFAS: American Orthopaedic Foot and Ankle Society; ROM: Rang of Motion; FAAM: Foot and Ankle Ability Measures; PPT, pressure pain threshold; MFS: Maryland Foot Score

### Quality assessment

**Fig 2:**
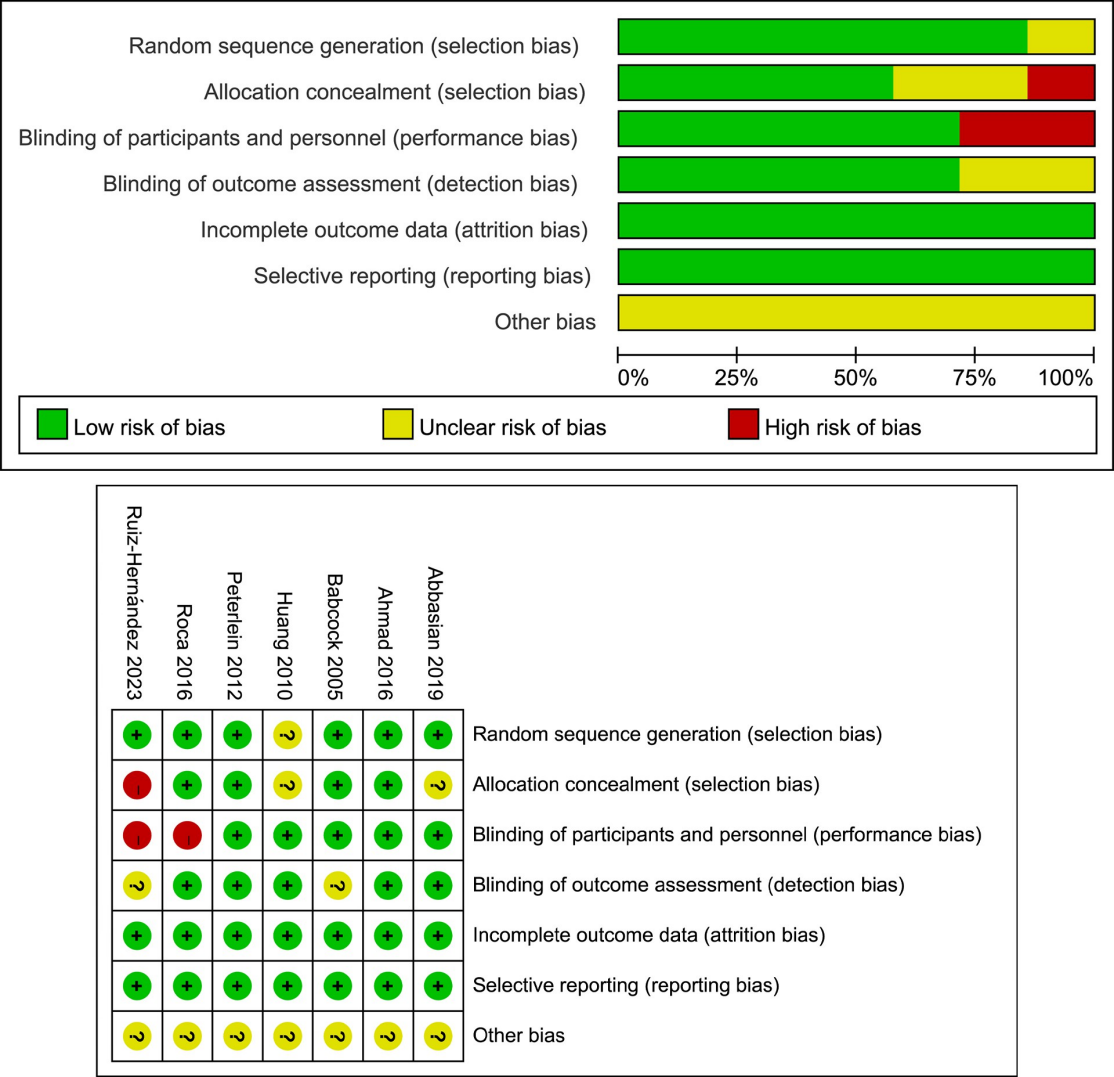
Risk of bias graph and summary.

The detailed quality assessment results were shown in **Fig 2**. Of the seven studies, only one [14] did not clearly report its randomization process. Allocation concealment was not described in two studies [14, 22] and high risk in another study [24]. Regarding the binding of participants, personnel, and outcome assessment, two studies [17, 24] had a high risk of bias. All studies were listed as low risk of bias for incomplete outcome data and selective reporting. However, concerning other potential sources of bias, the risk assessment remained unclear across all studies.

### Therapeutic efficacy

**Fig 3:**
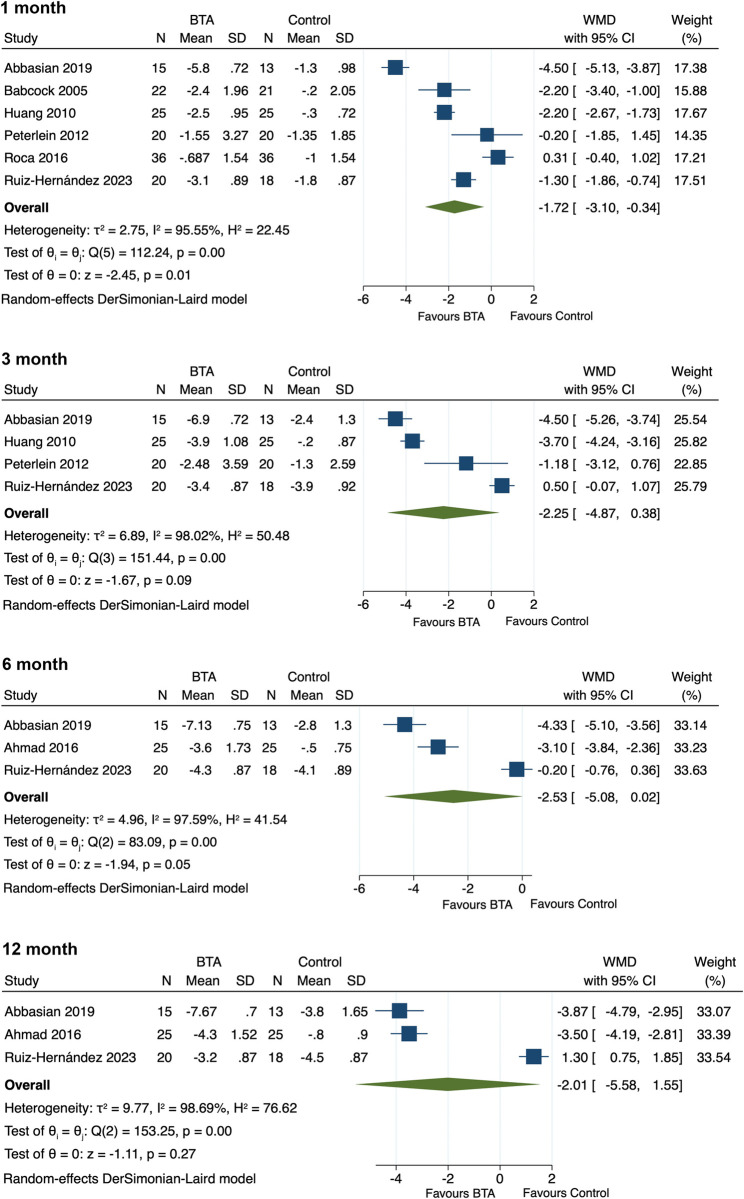
Forest plot of pain relief for comparing BTA with control interventions in patients with plantar fasciitis at 1-month, 3-month, 6-month and 12-month posttreatment.

All included studies evaluated the effects of BTA on pain reduction in PF patients, with the pooled results indicating a statistically significant pain score reduction in the BTA group at 1-month posttreatment compared to the control group (SMD = -1.72, 95% CI [-3.10, -0.34], p = 0.01]). No significant differences between groups were observed at 3-month, 6-month, and 12-month follow-ups. (3-month: SMD = -2.25, 95% CI [-4.87, 0.38], p = 0.09; 6-month: SMD = -2.53, 95% CI [-5.08, 0.02], p = 0.06; 12-month: WMD = -2.01, 95% CI [-5.58, 1.55], p = 0.27) (**Fig 3**).

**Fig 4:**
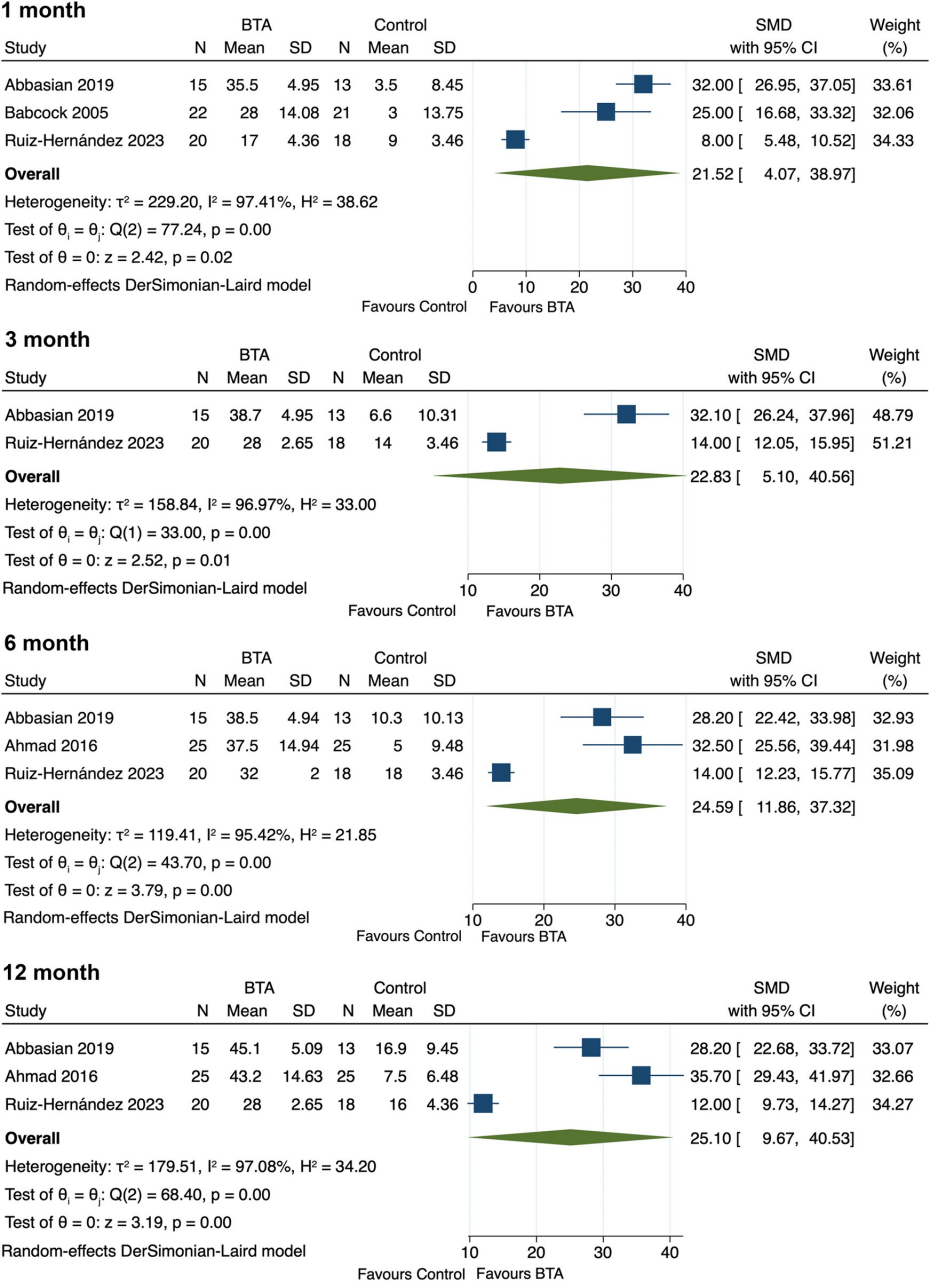
Forest plot of functional improvement for comparing BTA with control interventions in patients with plantar fasciitis at 1-month, 3-month, 6-month and 12-month posttreatment.

Four studies [13, 22–24] reported on the therapeutic effects of BTA injections on the functional status of PF patients, with data synthesis indicating beneficial effects at all follow-up times. (1-month: SMD = 21.52, 95% CI [4.07, 38.97], p = 0.02; 3-month: SMD = 22.83, 95% CI [5.10, 40.56], p = 0.01; 6-month: SMD = 24.59, 95% CI [11.86, 37.32], p<0.001; 12-month: SMD = 25.10, 95% CI [9.67, 40.53], p = 0.001) (**Fig 4**).

### Adverse events

**Fig 5:**
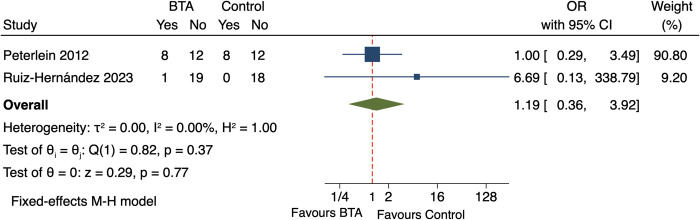
Forest plot of the incidence of adverse events for comparing BTA with control interventions in patients with plantar fasciitis.

No serious adverse events (AEs) were reported in any of the included studies. Only two studies [16, 24] reported treatment-related AEs, which were generally mild and temporary. Pooled analysis revealed no significant difference in the incidence of AEs between the BTA and control groups (OR = 1.19, 95% CI [0.36, 3.92], p = 0.37) (Fig 5).

## Discussion

To our knowledge, this study represented the first systematic review and meta-analysis to evaluate the efficacy and safety of botulinum toxin type A injections for treating plantar fasciitis. Seven randomized controlled trials involving 305 participants that met the selection criteria were identified. The pooled results indicated that BTA injections led to significant pain reduction in the short term (one month) and sustained functional improvement over twelve months compared to the control group, with no significant difference in adverse event occurrence between BTA and control interventions.

Botulinum toxin A was a neurotoxin produced by Clostridium botulinum, which was widely researched and applied in clinical settings for its muscle-paralyzing effects [25]. In recent years, an increasing body of evidence had emerged, supporting its efficacy in pain modulation [26]. The mechanisms underlying BTA’s effects on plantar fasciitis remained partially elucidated; however, studies indicated BTA might inhibit the reuptake of acetylcholine at presynaptic junctions, thereby inducing muscle weakness [27]. This reduction in muscle strength was believed to decrease tissue tension and subsequently ameliorate fascial or muscular-derived pain. BTA has been shown to exert analgesic effects through the partial suppression of neurotransmitters at sensory afferent terminals in a rat formalin model [28]. Furthermore, it facilitated the inhibition of peripheral sensitization, thereby indirectly contributing to a reduction in central sensitization [29]. These findings partially explained the analgesic action of BTA on plantar fasciitis, as demonstrated in the current study. However, previous studies provided limited information on the temporality of its analgesic effects, with the onset and duration of its efficacy for plantar fasciitis possibly varying from days to weeks. Based on our pooled results, BTA showed superior analgesic effects in the short term compared to the control group. This contrasted with the findings of another meta-analysis conducted by Acosta-Olivo et al. [30], which reported that BTA injections offer both short- and long-term efficacy (up to twelve months) in alleviating plantar heel pain. It is important to note that the control groups varied significantly among the included studies in their meta-analysis (corticosteroid, anesthetic, or normal saline), whereas our study exclusively included and analyzed RCTs that directly compared BTA with placebo injections. Such variation may account for the differing results between the two studies. Consistent with previous meta-analyses [30, 31], our findings also indicated significant functional improvements in plantar fasciitis patients following BTA injections, with effects lasting up to twelve months. While recent research suggested that functional recovery may be linked to changes in fascia thickness [32], the limited data available from the included studies did not allow further exploration of this relationship.

Our review demonstrated that the incidence of adverse events from BTA injections was comparable to the control group and aligned with the findings from other studies. The reported treatment-related adverse events included muscle weakness and injection-site pain, among others, but were generally mild and temporary. This evidence suggested that BTA injection can be considered a safe intervention for treating patients with plantar fasciitis. However, several limitations associated with this review should be noted. First, despite conducting a comprehensive literature search, only seven studies were identified and included in this meta-analysis. The limited number of studies may have reduced the statistical power to detect significant finding. Second, most of the included studies had methodological flaws due to the lack or weak descriptions of randomization methods, allocation concealment, and blinding. The possibility of biased results could not be ruled out, and thus, more well‐designed RCTs are required. Furthermore, variability in injection protocols and baseline characteristics of participants among the included studies could potentially influence the pooled effect of BTA. Consequently, additional research is necessary to explore the optimal management of BTA injections for patients with plantar fasciitis.

## Conclusion

In summary, the pooled data from seven eligible RCTs demonstrated that BTA injections resulted in significant short-term pain reduction and sustained functional improvement for patients with plantar fasciitis when compared to the control group, with no major adverse events reported in all included studies. These findings suggested that BTA injections could be an effective and safe therapeutic strategy for plantar fasciitis. Further large-scale, rigorously designed RCTs are needed to validate these findings and determine the optimal injection dosage and site for BTA in the treatment of plantar fasciitis.

## Supporting information

S1 Checklist PRISMA 2020 checklist. (DOCX)

S1 Table List of excluded studies with reasons. (DOCX)

S1 File Raw data for meta-analysis. (XLSX)

S2 File (XLS)
