# Supplementary material for: Therapeutic efficacy and safety of botulinum toxin A injection in plantar fasciitis: A systematic review and meta-analysis
Source: PLoS One. 2024 Dec 17;19(12):e0312908. doi: 10.1371/journal.pone.0312908 (PMC11651609; doi:10.1371/journal.pone.0312908)
Supplement: S1 Table — (DOCX) [file pone.0312908.s002.docx]

**S1 Table. List of excluded studies with reasons**

| Study | Title | Reason for exclusion |
| --- | --- | --- |
| Poenaru D, 2023 | Pain Modulation in Chronic Musculoskeletal Disorders: Botulinum Toxin, a Descriptive Analysis. | Not RCT |
| Assad, 2016 | Novel and Conservative Approaches Towards Effective Management of Plantar Fasciitis. | Not RCT |
| Li TT, 2023 | Clinical efficacy of botulinum toxin type A in the treatment of fasciitis pain: A systematic review and meta-analysis. | Not RCT |
| Molloy LA, 2012 | Managing chronic plantar fasciitis: when conservative strategies fail. | Not RCT |
| Tsikopoulos K, 2016 | Injection therapies for plantar fasciopathy ('plantar fasciitis'): a systematic review and network meta-analysis | Not RCT |
| Acosta-Olivo C, 2022 | Clinical Efficacy of Botulinum Toxin in the Treatment of Plantar Fasciitis: A Systematic Review and Meta-analysis of Randomized Controlled Trials. | Not RCT |
| Li H, 2018 | Comparison of efficacy of eight treatments for plantar fasciitis: A network meta-analysis. | Not RCT |
| Seyler TM, 2008 | Botulinum neurotoxin as a therapeutic modality in orthopaedic surgery: more than twenty years of experience. | Not RCT |
| Zhang T, 2011 | The efficacy of botulinum toxin type A in managing chronic musculoskeletal pain: a systematic review and meta analysis. | Not RCT |
| Gao R, 2021 | Comparative Effectiveness of Minimally Invasive Nonsurgical Treatments for Plantar Fasciitis: A Network Meta-analysis of 30 Randomized Controlled Trials. | Not RCT |
| Al-Boloushi Z, 2019 | Minimally invasive non-surgical management of plantar fasciitis: A systematic review. | Not RCT |
| Cotchett MP, 2010 | Effectiveness of dry needling and injections of myofascial trigger points associated with plantar heel pain: a systematic review. | Not RCT |
| Placzek R, 2005 | Treatment of chronic plantar fasciitis with botulinum toxin A: an open case series with a 1 year follow up. | Not RCT |
| Chou LW, 2011 | Serial ultrasonographic findings of plantar fasciitis after treatment with botulinum toxin a: a case study | Not RCT |
| Placzek R, 2006 | Treatment of chronic plantar fasciitis with botulinum toxin A--an open pilot study on 25 patients with a 14-week-follow-up | Not RCT |
| Orthop, 2005 | Botulinum toxin A--therapy option in cases of chronic plantar fasciitis?-an open treatment attempt with 9 patients and a one year observation period | Not RCT |
| Gill LH, 1997 | Diagnosis and Conservative Management. | Not RCT |
| Lynch DM, 1998 | Conservative treatment of plantar fasciitis. A prospective study. | Not RCT |
| Singh JA, 2013 | Use of botulinum toxin in musculoskeletal pain. | Lacking a clear diagnosis of plantar fasciitis |
| Rawicki B, 2010 | Botulinum toxin assessment, intervention and aftercare for paediatric and adult niche indications including pain: international consensus statement. | Lacking a clear diagnosis of plantar fasciitis |
| Jabbari B, 2011 | Treatment of refractory pain with botulinum toxins—an evidence-based review. | Lacking a clear diagnosis of plantar fasciitis |
| Radovic P, 2020 | Treatment of "plantar fasciitis"/Plantar Heel Pain Syndrome with botulinum toxin - A novel injection paradigm pilot study. | Lacking a clear diagnosis of plantar fasciitis |
| Jabbari B, 2008 | Botulinum neurotoxins in the treatment of refractory pain. | Lacking a clear diagnosis of plantar fasciitis |
| Bruno VA, 2016 | Botulinum Toxin Use in Refractory Pain and Other Symptoms in Parkinsonism. | Lacking a clear diagnosis of plantar fasciitis |
| Monto RR, 2013 | Platelet-rich plasma and plantar fasciitis. | Combined with other interventions |
| Elizondo-Rodríguez J, 2021 | Comparison of Botulinum Toxin A, Corticosteroid, and Anesthetic Injection for Plantar Fasciitis. | Combined with other interventions |
| Dı ´az-Llopis IV, 2012 | Randomized controlled study of the efficacy of the injection of botulinum toxin type A versus corticosteroids in chronic plantar fasciitis: results at one and six months | Combined with other interventions |
| Ahadi T, 2022 | Comparison of the Effect of Ultrasound-Guided Injection of Botulinum Toxin Type A and Corticosteroid in the Treatment of Chronic Plantar Fasciitis: A Randomized Controlled Trial. | Combined with other interventions |
| Elizondo-Rodriguez J, 2013 | A comparison of botulinum toxin a and intralesional steroids for the treatment of plantar fasciitis: a randomized, double-blinded study. | Combined with other interventions |
| ﻿Dı ´az-Llopis IV, 2013 | Botulinum toxin type A in chronic plantar fasciitis: clinical effects one year after injection. | Having incomplete data |
| Logan LR, 2006 | Autologous blood injection and botulinum toxin for resistant plantar fasciitis accompanied by spasticity. | Having incomplete data |
| Placzek R, 2006 | Treatment of chronic plantar fasciitis with Botulinum toxin A: preliminary clinical results. | Having incomplete data |
